# Knee Cartilage Thickness, T1ρ and T2 Relaxation Time Are Related to Articular Cartilage Loading in Healthy Adults

**DOI:** 10.1371/journal.pone.0170002

**Published:** 2017-01-11

**Authors:** Sam Van Rossom, Colin Robert Smith, Lianne Zevenbergen, Darryl Gerard Thelen, Benedicte Vanwanseele, Dieter Van Assche, Ilse Jonkers

**Affiliations:** 1 Human movement biomechanics research group, Department of kinesiology, Katholieke Universiteit Leuven, Leuven, Belgium; 2 Department of mechanical engineering, University of Wisconsin-Madison, Madison, United States of America; 3 Department of biomedical engineering, University of Wisconsin-Madison, Madison, United States of America; 4 Department of orthopedics and rehabilitation, University of Wisconsin-Madison, Madison, United States of America; 5 Musculoskeletal rehabilitation research group, Department of rehabilitation sciences, Katholieke Universiteit Leuven, Leuven, Belgium; Semmelweis Egyetem, HUNGARY

## Abstract

Cartilage is responsive to the loading imposed during cyclic routine activities. However, the local relation between cartilage in terms of thickness distribution and biochemical composition and the local contact pressure during walking has not been established. The objective of this study was to evaluate the relation between cartilage thickness, proteoglycan and collagen concentration in the knee joint and knee loading in terms of contact forces and pressure during walking. 3D gait analysis and MRI (3D-FSE, T1ρ relaxation time and T2 relaxation time sequence) of fifteen healthy subjects were acquired. Experimental gait data was processed using musculoskeletal modeling to calculate the contact forces, impulses and pressure distribution in the tibiofemoral joint. Correlates to local cartilage thickness and mean T1ρ and T2 relaxation times of the weight-bearing area of the femoral condyles were examined. Local thickness was significantly correlated with local pressure: medial thickness was correlated with medial condyle contact pressure and contact force, and lateral condyle thickness was correlated with lateral condyle contact pressure and contact force during stance. Furthermore, average T1ρ and T2 relaxation time correlated significantly with the peak contact forces and impulses. Increased T1ρ relaxation time correlated with increased shear loading, decreased T1ρ and T2 relaxation time correlated with increased compressive forces and pressures. Thicker cartilage was correlated with higher condylar loading during walking, suggesting that cartilage thickness is increased in those areas experiencing higher loading during a cyclic activity such as gait. Furthermore, the proteoglycan and collagen concentration and orientation derived from T1ρ and T2 relaxation measures were related to loading.

## Introduction

Healthy cartilage is essential for optimal joint function as it distributes loading and reduces friction between articulating bones. Mechanical factors are known to influence cartilage homeostasis, and are therefore essential for the maintenance of cartilage health[1–3]. In order to better understand the role of loading on the pathomechanics of degenerative cartilage diseases, such as osteoarthritis (OA), it is important to understand the influence of mechanical factors on the thickness and composition of cartilage.

Cartilage thickness has been used as an in-vivo measure of cartilage health. Previous research found that cartilage adapts to chronic loading patterns occurring during walking[4]. Increased cartilage volume was found with increased physical activity level in healthy children and with increased muscle cross-sectional area in healthy adults[5,6]. A positive correlation between knee external adduction moment (KAM) and medial cartilage thickness or medial to lateral thickness ratio in a cohort of healthy adults was reported [7–9]. Knee and shoulder cartilage thinning was observed in paraplegic patients due to unloading[10,11]. This shows that cartilage is sensitive to mechanical stimuli during routine daily life activities. Additionally, it was suggested that cyclic and repetitive loading patterns, during walking in particular, dominate the biologic and structural response of cartilage[8]. Indeed, knee flexion angle at heel strike was correlated with the thickness distribution of the medial femur condyle and the thickest region of cartilage was found to coincide with the contact region at heel strike[8,12]. Therefore, they hypothesized that cartilage was thicker in the load-bearing regions of the knee as a long term adaptation to the high compressive forces at heel strike [4,12,13].

In the past, external joint moments were used to estimate knee loading and to analyze the relationship with cartilage thickness. However, as external moments represent the combined effects of muscle, ligament and cartilage contact forces, they do not explicitly characterize the internal loads acting on the cartilage. While these forces cannot be measured in-vivo, novel musculoskeletal modeling techniques allow estimation of muscle and ligament forces and consequently knee contact forces and even local contact pressure[14,15]. As a result, a more detailed description of joint loading can now be provided to explore the correlation with local cartilage structural properties.

Structural changes in cartilage are often preceded by changes in biochemical composition[16–18]. Initial cartilage deterioration induces loss of proteoglycans and increases in water content in combination with disorganization and loss of the collagen matrix[19]. Advancements in magnetic resonance (MR) imaging and more specific T1ρ and T2 mapping have been used to identify these early changes in matrix-composition. Increased T1ρ relaxation time, as observed in OA patients, is related to proteoglycan loss, whereas increased T2 relaxation time is related to collagen deterioration and disorganization[20–24]. These relaxation times can therefore be used to evaluate cartilage condition and biologic response to loading. Indirect estimates of loading were previously used to evaluate the relation with cartilage composition. No differences in T1ρ or T2 relaxation times between healthy active and sedentary adults were found[22]. Six weeks of unloading resulted in significantly increased T1ρ and T2 relaxation times, suggesting that changes in the biochemical composition result from unloading. However T1ρ and T2 values were restored to baseline after 4 weeks of weight-bearing[25]. A decrease in T2 relaxation time was found after a standardized training period [26,27]. T1ρ relaxation times were increased after running a marathon[28]. This showed that loading could possibly modify cartilage composition. A higher ratio of the quadriceps medial to lateral cross-sectional area was found to relate to higher frontal plane loading during walking and higher T1ρ and T2 relaxation times for the whole joint complex, however the relation between frontal plane loading and T1ρ and T2 relaxation times was not directly tested[29]. Thus, there is no confirmed association between cartilage composition and increased frontal plane loading. Acute compressive loading resulted in a significant decreased T1ρ and T2 relaxation time of the medial condyle in healthy adults[30]. Furthermore, T1ρ and T2 relaxation times were found to be lower in healthy persons subjected to higher sagittal plane moments during a drop jump, suggesting a protective response of cartilage to loading[31]. However healthy subjects with higher KAM, and thus increased frontal plane loading during the drop jump presented elevated T1ρ values in the medial compartment compared to the lateral compartment[31]. These in-vivo findings suggest that joint loads exceeding the physiological weight-bearing capacity of cartilage might induce cartilage degeneration[32]. In support of these in-vivo findings, in-vitro studies also demonstrated that mechanical loading can promote either synthesis or breakdown of the cartilage components depending on loading regimes[33,34]. Furthermore, animal experiments showed that the cartilage composition and mechanical properties adapt to mechanical stimulation[35]. Although indirect evidence exists that cartilage thickness and biochemical composition relate to loading, none of these studies related structural and biochemical outcome parameters to local cartilage loading in a whole joint complex during functional activities.

This exploratory study relates local femoral cartilage thickness and biochemical composition to cartilage tissue loading during walking. MR imaging was used to measure cartilage thickness, proteoglycan and collagen content, while a multibody musculoskeletal model was used to estimate cartilage contact pressures. Higher cartilage loading is expected to relate to increased thickness and to lower T1ρ and T2 relaxation times, indicative for a higher proteoglycan and collagen concentration and orientation.

## Materials and Methods

### Subjects

**Fig 1:**
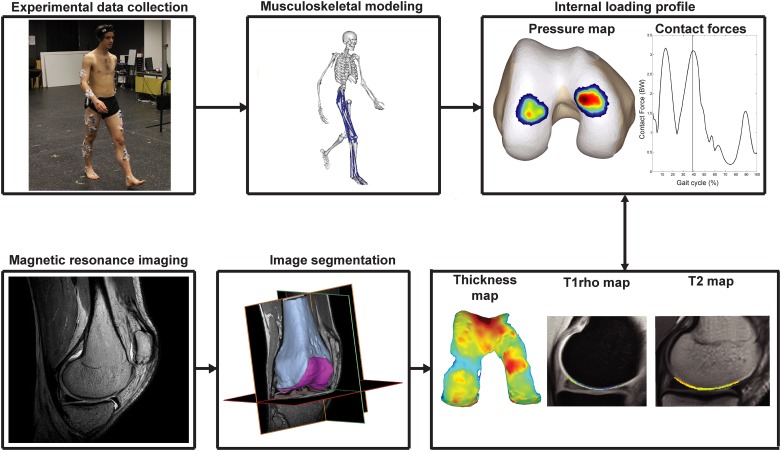
Schematic overview of the workflow. Experimental gait data was collected and processed using musculoskeletal modeling in order to calculate the cartilage contact force and pressure distribution. High resolution MR-images were captured and segmented to calculate thickness maps and to outline the cartilage on the T1ρ and T2 maps. Loading parameters were correlated with the peak and mean thickness, mean T1ρ relaxation time and mean T2 relaxation time to explore the relation between localized loading and cartilage thickness and composition.

Fifteen healthy subjects, with no history of knee injuries were recruited to participate in the current study (Table 1). Experimental motion analysis data and MR-images of all participants were acquired on the same day (mean time between motion analysis and MR-acquisition = 2h). An overview of the overall study design is provided in Fig 1. All procedures were approved by the university hospital Leuven ethics committee (s56093) and informed written consent was obtained from all participants.

**Table 1:** Patient characteristics.

Demographics	
Gender	8 Male/7 Female
Weight	70.49 ± 7.24 kg
Height	1.77 ± 0.06 m
Age	30.73 ± 5.84 years
Dominant leg	13 Right /2 Left
Walking speed	1.39 ± 0.12 m/s
First peak GRF	765.07 ± 85.93 N
Second peak GRF	791.55 ± 86.01 N
Alignment*	183.87 ± 2.18°
Leg difference	0.0068 ± 0.0044 m

*Alignment of the anatomical axis was determined on lying MRI, values >180° indicate valgus.

### Motion analysis

#### Data collection

A 10-camera Vicon system (Vicon, Oxford Metrics 100Hz) was used to capture three-dimensional marker positions during gait. Synchronously, ground reaction forces were captured using two force plates embedded in the walkway (AMTI, Watertown, USA, 1000Hz). Retro-reflective markers were placed according to an extended Plug-in-Gait marker set (S1 Fig)[36]. After a static calibration trial, participants were instructed to walk barefoot at their habitual walking speed (mean walking speed = 1.39 ± 0.12 m/s, range 1.19–1.59m/s) across the motion lab (8m). Three trials with valid force plate contact were captured and retained for further processing. Ground reaction forces were filtered using a second order Butterworth low pass filter, with cut-off level at 30Hz and marker positions were filtered using a smoothing spline with cut-off at 6Hz before entering the musculoskeletal modeling workflow.

#### Musculoskeletal modeling

Muscle and knee contact forces were estimated using a scaled 3D musculoskeletal model that has been presented previously[37]. A customized knee joint allowing 6 degrees of freedom (DoF) tibiofemoral and patellofemoral joint kinematics was implemented in a generic lower extremity model[38]. The model included 44 musculotendon actuators spanning the right hip, knee and ankle. Additionally, 14 bundles of non-linear springs represented the major knee ligaments and capsule. Cartilage contact pressure was calculated using a non-linear elastic foundation formulation that calculates the local contact pressure based on the penetration depth between overlapping cartilage surface meshes[39]. Uniform cartilage thickness distribution was assumed in both joints, with a combined thickness of 4mm and 7mm in the tibiofemoral and patellofemoral joint respectively. An elastic modulus of 10MPa and a Poisson’s ratio of 0.45 was defined for the cartilage[40,41]. The lower extremity model was implemented in SIMM with the Dynamics Pipeline (Musculographics Inc., Santa Rosa, CA) and SD/Fast (Parametric Technology Corp., Needham, MA) used to generate the multibody equations of motion[42].

After scaling the generic model to the subjects’ anthropometry, joint angles were calculated using inverse kinematics[43]. Next, the muscle activations and secondary knee kinematics (11 DoF), required to reproduce the measured primary hip, knee and ankle accelerations were computed using the concurrent optimization of muscle activations and kinematics (COMAK) algorithm[39]. Only the knee flexion angle was prescribed during the optimization, while secondary tibiofemoral and all patellofemoral DoF evolved as a function of muscle, ligament and contact forces[37,39,44].

For each trial, the timing of the two peaks of the resultant tibiofemoral contact force during the stance phase was determined and the coinciding mean and maximal contact pressures, contact areas, as well as the components of the contact forces expressed in the femur reference frame were analyzed. Furthermore, the impulse of the contact forces and the average contact pressure over the whole stance phase was calculated. These load describing parameters were not normalized to bodyweight nor dimensions, as this represents a more physiological estimate of tissue level loading. All variables were analyzed for the medial, lateral and combined femoral condyle(s) for each trial and then averaged over the three trials.

### Medical imaging

Imaging of the dominant leg was performed on a 3T Ingenia scanner, with a standard transmit and receive knee coil (Philips Healthcare, Best, The Netherlands). After one hour standardized rest in order to eliminate the influence of previous loading, participants were positioned in supine position with the knee in neutral internal rotation and full extension. During the scans the knee was fixated to minimize movement. The following scanning sequences were acquired: 1) a high resolution 3D-fast spin echo acquisition (3D-FSE), 2) T1ρ relaxation time sequence and 3) T2 relaxation time sequence. MRI sequence parameters are listed in Table 2.

**Table 2:** Overview of the MRI sequence parameters.

	3D-FSE	T1ρ	T2
TR (ms) / ET (ms)	1800/120	5.9587/3.082	4000/11-22-33-44-55-66-77-88
Field of view (cm)	16	16	16
Matrix	268 x 268	292 x 256	160 x 160
Slice thickness (mm)	1	4	4
Echo train length	85	64	12
Bandwidth (kHz)	562	522	367
Number of excitations	2	1	1
Number of slices	320	20	20
Acquisition time (min)	5.94	17.20	5.24
Time of recovery (ms)	/	2000	/
Time of spinlock (ms)	/	0/10/20/40/60	/
Frequency of spinlock (Hz)	/	500	/

The femoral cartilage and distal part of the femur were manually segmented by the same author (SVR) from the 3D high-resolution images (Mimics Innovation Suite, Materialise, Leuven, Belgium). 3D triangulated surfaces of the distal femur (subchondral bone) and the femoral cartilage were created. Next, a cartilage thickness distribution map was generated by computing the minimum distance between the subchondral bone surface and the cartilage surface for every vertex of the cartilage surface in the surface normal direction[45]. The average and peak cartilage thickness was calculated for the weight-bearing area of the medial and the lateral condyle. The weight-bearing area was defined as the area between the anterior end of the intercondylar notch and 60% of the distance to the most posterior end of the femoral condyles[46,47]. The peak thickness of the weight-bearing zone was defined as the mean of the top 10% of all thickness values in the weight-bearing zone, whereas the mean thickness was calculated using all thickness values of the weight-bearing region[45]. Next, the subject-specific thickness maps were anisotropically registered to the generic cartilage mesh used in the musculoskeletal model (S1 Text). The 3D volumetric meshes were registered on the T1ρ and T2 images to outline the cartilage regions of interest. Relaxation time maps for the T1ρ and T2 sequences were generated using a pixel-by-pixel based evaluation of the mono-exponential Levenberg-Marquardt fitting algorithm[48]:

$$M\left(TSL\right)\propto \text{exp}\left(-\frac{TSL}{T1\rho }\right)$$

$$M\left(TE\right)\propto \text{exp}\left(-\frac{TE}{T2}\right)$$

Voxels with a T1ρ relaxation time > 130ms or T2 relaxation time >100ms were excluded to avoid artifacts due to partial volume effects with synovial fluid[49]. An average T1ρ and T2 relaxation time was calculated for the weight-bearing part of the medial and lateral femur condyle separately and for both condyles together. T1ρ values of one single subject were excluded from the analysis due to image artifacts, resulting in higher relaxation times.

### Statistical analysis

Differences between the medial and lateral condyle load describing parameters, mean and peak thickness, T1ρ and T2 relaxation time were tested by a Wilcoxon paired-samples test. The average and peak thickness of the weight bearing zone of the medial and lateral condyle were correlated to the different loading variables using a one-tailed Spearman rank correlation coefficient. Furthermore, the correlation between thickness and the local pressure was calculated for every individual mesh face in contact, resulting in a correlation map indicative of the relation between local thickness and loading in the respective contact regions. The relation between cartilage composition and loading was analyzed by correlating the average relaxation times of the weight-bearing zone of the total knee and of the medial and lateral condyle to their respective loading variables, using a two-tailed Spearman rank correlation coefficient. Significance level was set at p = 0.05 for all conducted statistical tests in MATLAB (MATLAB 2012b, The Math Works, Inc., Natick, Massachusetts, USA).

## Results

The average and standard deviations of all loading variables are summarized in Table 3. Contact areas on the medial condyle were on average 251 ± 25mm^2^ and 279 ± 36mm^2^ during the first and second peak, respectively. Contact areas on the lateral condyle were on average 177 ± 14mm^2^ and 215 ± 40mm^2^ for the first and second peak, respectively. Medial resultant contact forces were 1308 ± 205N and 1399 ± 214N for the first and second peak, respectively and were significantly higher than the lateral resultant contact forces, which were 885 ± 260N and 698 ± 138N for the first and second peak, respectively (Table 3). Mean and maximal pressures on the medial condyle at the first peak were not significantly higher than the pressures on the lateral condyle (6.01–12.75MPa and 5.80–12.49MPa for the medial and lateral mean and peak pressures, respectively) (Table 3). In contrast, mean and maximal pressures on the medial condyle at the second peak were significantly higher than the pressures on the lateral condyle (6.12–12.17MPa and 4.21–8.67MPa for the medial and lateral mean and peak pressures, respectively). Mean thickness of the medial and lateral condyle was on average 2.70 ± 0.38mm and 2.44 ± 0.28mm, respectively. Average peak thickness was 3.77 ± 0.57mm and 3.46 ± 0.38mm for the medial and lateral condyle, respectively. The weight-bearing part of the medial condyle was significantly thicker compared to the lateral condyle (P = 0.0034 and P = 0.03 for the mean and peak thickness, respectively). Medial and lateral T1ρ relaxation times were on average 41.59 ± 5.49ms and 46.23 ± 7.18ms, respectively. T1ρ relaxation time of the medial condyle was significantly lower compared to the lateral condyle (P = 0.004). Medial and lateral average T2 relaxation times were 59.42 ± 7.18ms and 57.41 ± 10.30ms, respectively.

**Table 3:** Average and standard deviations of all loading variables.

	Total knee	Medial condyle	Lateral condyle	
	Average ± Deviation	Average ± Deviation	Average ± Deviation	P-value
**Mean Pressure [MPa]**				
First peak	5.97 ± 0.74	6.01 ± 0.57	5.8 ± 1.32	0.3028
Second peak	5.35 ± 0.5	6.12 ± 0.72	4.21 ± 0.54	0.0001*
**Maximal Pressure [MPa]**				
First peak	13.93 ± 2.08	12.75 ± 1.54	12.49 ± 2.91	0.4887
Second peak	12.26 ± 1.25	12.17 ± 1.39	8.67 ± 1.29	0.0001*
**Average Pressure during Stance [MPa]**	3.648 ± 0.275	3.981 ± 0.424	3.211 ± 0.283	0.0002*
**First peak contact force [N]**				
Anterior-Posterior	483.95 ± 201.99	344.39 ± 128.64	139.56 ± 86.26	0.0001*
Compression	2062.35 ± 308.01	1230.65 ± 201.57	831.7 ± 247.3	0.0012*
Medial-lateral	10.81 ± 46.98	-248.92 ± 31.03	259.72 ± 60.49	0.6387
Resultant	2125.59 ± 328.56	1308.46 ± 204.86	885.28 ± 259.87	0.0006*
**Second peak contact force [N]**				
Anterior-Posterior	11.19 ± 167.09	-18.47 ± 120.51	29.66 ± 50.19	0.0084*
Compression	2012.64 ± 290.35	1355.55 ± 216.19	657.09 ± 132.02	0.0001*
Medial-lateral	-94.52 ± 28.35	-315.87 ± 64.06	221.35 ± 56.21	0.0001*
Resultant	2023.62 ± 279.35	1399.47 ± 213.91	697.54 ± 138.47	0.0001*
Impulse [N*s]				
Anterior-Posterior	86.28 ± 54.8	51.48 ± 31.79	34.81 ± 24.3	0.0004*
Compression	863.74 ± 108.26	536.69 ± 96.99	327.04 ± 52.92	0.0001*
Medial-lateral	-22.1 ± 7.57	-125.63 ± 18.39	103.53 ± 16.52	0.0001*
Resultant	875.7 ± 104.07	559.92 ± 94.88	347.24 ± 54.28	0.0001*

* indicates a significant difference between medial and lateral loading (p < 0.05)

Mean and peak thickness of the lateral and medial condyle correlated significantly with the load describing parameters (Table 4). Mean and peak thickness of both the medial and lateral condyle correlated significantly with the compressive and resultant contact force of the total knee. Likewise, the mean and peak thickness of the medial condyle correlated significantly with the compressive and resultant contact force on the medial condyle at second peak. However, these correlations were not confirmed for the lateral condyle. Nevertheless, the mean and peak lateral thickness correlated significantly with the lateral condyle impulses. In line with the correlations found for the contact forces, the mean and peak cartilage thickness of both condyles as well as the medial and lateral condyle thickness correlated significantly with the average pressure on the total knee as well as on the medial and lateral condyle, respectively. Scatterplots of the significant correlations are provided in supplementary material (S2, S3, S4 and S5 Figs).

**Table 4:** Significant correlations between cartilage thickness and the loading parameters. Spearman correlation coefficient and p-value are given.

	Medial thickness		Lateral thickness	
	Mean	Peak	Mean	Peak
**Total knee**				
First peak contact force				
*Anterior-posterior*	0.48 (0.036)	n.s	0.55 (0.019)	0.55 (0.019)
*Compression*	0.45 (0.047)	n.s	n.s	n.s
Second peak contact force				
*Compression*	0.62 (0.008)	0.57 (0.014)	0.57 (0.015)	n.s
*Resultant*	0.62 (0.008)	0.57 (0.014)	0.57 (0.015)	n.s
Average pressure during stance	0.78 (0.001)	0.73 (0.001)	0.55 (0.019)	n.s
**Medial Condyle**				
Second peak contact force				
*Compression*	0.55 (0,018)	0.60 (0.010)	n.a.	n.a.
*Resultant*	0.54 (0,020)	0.56 (0.017)	n.a.	n.a.
Average pressure during stance	0.58 (0,014)	0.71 (0.002)	n.a.	n.a.
**Lateral Condyle**				
Impulse				
*Anterior-posterior*	n.a.	n.a.	n.s	0.50 (0.030)
*Compression*	n.a.	n.a.	0.46 (0.043)	0.50 (0.029)
*Medial-lateral*	n.a.	n.a.	0.53 (0.024)	n.s
*Resultant*	n.a.	n.a.	0.46 (0.043)	0.50 (0.029)
Average pressure during stance	n.a.	n.a.	0.57 (0.015)	0.71 (0.002)
First peak mean pressure	n.a.	n.a.	n.s	0.49 (0.032)
First peak max pressure	n.a.	n.a.	n.s	0.50 (0.031)

n.s.: not significant, n.a.: not applicable

**Fig 2:**
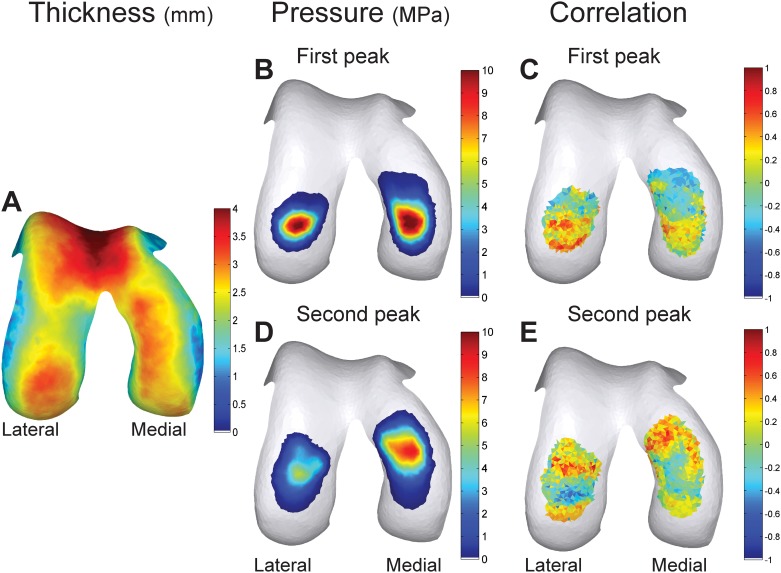
Local correlations between cartilage pressure and thickness. (A) Average thickness distribution of all subjects, (B) Average pressure map of the first peak, (D) Average pressure map of the second peak. (C & E) Correlation map of the correlations between the mesh face specific thickness and pressure. (C) Shows the correlations at the first peak, (E) shows the correlations at the second peak.

The correlation map (Fig 2) showed that the area where local cartilage thickness correlated with local pressure was larger for the lateral condyle. For the medial condyle, 5.13% of the contact area at the first peak (12.87 mm^2^) and 20.67% of the contact area at the second peak (57.75 mm^2^) had a thickness that was significantly correlated with its local pressure (mean R = 0.49 ± 0.05, range: 0.44–0.58 and mean R = 0.53 ± 0.07, range: 0.44–0.79 at the first and second peak, respectively). For the lateral condyle, 30.56% of the contact area at the first peak (54.06 mm^2^) and 17.86% of the contact area at the second peak (38.48 mm^2^) had a thickness that was significantly correlated with its local pressure (mean R = 0.55 ± 0.07, range: 0.45–0.71 and mean R = 0.57 ± 0.1, range: 0.45–0.83 at the first and second peak, respectively).

Significant correlations between the average T1ρ relaxation times and load describing parameters were found (Table 5). The average whole joint T1ρ relaxation time was significantly correlated with the total knee impulses and contact forces at second peak. The average whole joint T2 relaxation time was significantly correlated with the second peak total knee compressive contact force (R = -0.53, P = 0.047), second peak total knee resultant contact force (R = -0.53, P = 0.047) and average total pressure during stance (R = -0.59, P = 0.020). Furthermore, the relation between total knee loading and average whole joint T1ρ relaxation time was confirmed at compartmental level: the average medial T1ρ relaxation time was significantly correlated with the medial contact force at second peak and with the medial condyle impulse, whereas the average lateral T1ρ relaxation time was significantly correlated with the total knee and lateral condyle contact forces at second peak. A full overview of the correlations is presented in S1, S2 and S3 Tables.

**Table 5:** Significant correlations between average T1ρ relaxation time and the loading parameters. Spearman correlation coefficient and p-value are given.

	Average T1ρ relaxation time		
	Total	Medial	Lateral
**Total knee**			
First peak contact force			
*Anterior -posterior*	n.s.	0.56 (0.042)	n.s.
Second peak contact force			
*Anterior -posterior*	0.66 (0.013)	n.s.	0.68 (0.010)
*Compression*	-0.55 (0.043)	n.s.	-0.59 (0.030)
*Resultant*	-0.55 (0.043)	n.s.	-0.59 (0.030)
Impulse			
*Anterior -posterior*	0.56 (0.042)	n.s.	0.71 (0.006)
*Compression*	-0.56 (0.042)	n.s.	n.s.
*Resultant*	-0.54 (0.048)	n.s.	n.s.
**Medial condyle**			
Second peak contact force			
*Medial-lateral*	n.a.	0.62 (0.021)	n.a.
Impulse			
*Anterior -posterior*	n.a.	0.69 (0.008)	n.a.
*Medial-lateral*	n.a.	0.75 (0.003)	n.a.
**Lateral condyle**			
Second peak contact force			
*Anterior -posterior*	n.a.	n.a.	0.70 (0.007)
*Medial-lateral*	n.a.	n.a.	-0.66 (0.012)

n.s.: not significant, n.a.: not applicable

## Discussion

The present study examined if femoral cartilage thickness and biochemical composition, measured by magnetic resonance imaging correlated with knee loading during walking, as calculated using musculoskeletal modeling. The results indicate that thicker cartilage on both the medial and lateral condyle was related to higher loading in the medial and lateral compartment, with higher T1ρ relaxation time being related to increased shear loading and lower T1ρ and T2 relaxation time being related to increased compression. This paper is, to the best of our knowledge, the first to relate local cartilage thickness and T1ρ and T2 relaxation times to the local mechanical loading at the articular surface.

In agreement with previous research, this study found a significant correlation between mean and peak thickness of the weight-bearing region of both condyles and overall cartilage loading (S2 and S3 Figs)[7–9]. Due to the specific methodology used i.e. a musculoskeletal model allowing the separate calculation of lateral and medial contact forces and pressures, local condyle thickness was found to relate to local condyle loading (S2 and S3 Figs). These findings suggest that the thickness of cartilage may be adapted to the cyclic loads experienced during ambulation. Medial condyle thickness distribution was mostly related to the second peak loading, whereas lateral condyle thickness was mostly related to the first peak loading. The fact that lateral condyle loading was highest at the first peak compared to the second peak can possibly explain this finding[50,51]. Local lateral condyle thickness distribution was more correlated to its local pressure, possibly because the lateral contact area was smaller compared to the medial contact area[8,12]. This resulted in a more isolated effect of loading in one region, whereas the effect of loading was more distributed on the medial condyle.

Previous research demonstrated a relation between KAM and medial condyle thickness, whereas we are first to describe the relation between cartilage thickness and cartilage loading of both condyles[7–9]. More specific, an additional relation between lateral condyle loading and lateral condyle thickness was found. This can be explained by the use of a more detailed estimate of internal joint loading, provided by musculoskeletal modeling, which takes muscle and ligament forces into account to determine joint loading, instead of using external estimates of joint loading as KAM. Indeed, KAM is an estimate of the ratio of loading between the medial and lateral condyles and does not accurately capture the magnitude of lateral compartment cartilage loading[52,53]. As a consequence, only a relation between an estimation of medial loading and medial thickness was previously found and confirmed by our findings.

Besides the relation with cartilage thickness, biochemical composition estimated by T1ρ and T2 relaxation times was also related to cartilage contact forces and pressures during gait. In the past, indirect estimates of loading were related to cartilage T1ρ and T2 relaxation times[29–31]. Results suggested that changes in biochemical composition occur secondary to loading, as reflected in the increased T1ρ relaxation time after unloading or running a marathon[25,28]. Furthermore lower T1ρ and T2 relaxation times were found in healthy persons subjected to higher sagittal plane moments during a drop jump[31]. Since a drop jump is performed less frequently compared to walking, one could expect a stronger relation between biochemical composition and loads during a more habitual cyclic activity such as walking. The present results further reinforce these insights and indicate that T1ρ and T2 relaxation time can be related to loading.

Surprisingly, direction of the force seems to be an important determinant of cartilage composition: Higher T1ρ relaxation times were related to anterior-posterior shear loading (S4 and S5 Figs), whereas lower T1ρ relaxation times were related to medial-lateral contact forces (S4 and S5 Figs). In contrast, both lower T1ρ and T2 relaxation times were related to higher compressive forces and pressures (S4 and S5 Figs). T1ρ relaxation time is related to cartilage proteoglycan concentration and T2 relaxation time is related to cartilage collagen concentration and orientation[20–24]. This suggests that in the presence of higher compressive forces, upregulation of the proteoglycan and collagen synthesis in the extracellular matrix may occur as a chronic response to loading due to a higher mechanical stimulation of the chondrocytes[33,34]. This finding is comparable to the decreased T2 relaxation times found after a standardized training period, suggesting adaptations of the extracellular matrix due to the imposed mechanical loading[26,27]. Similarly higher shear forces are known to accelerate cartilage deterioration and can explain the lower proteoglycan concentration, reflected as higher T1ρ relaxation time[33,34]. Next to loading magnitude and direction, loading time is an important factor explaining the inter-subject differences in T1ρ relaxation time: As a result, correlations between T1ρ relaxation time and impulses of the contact force were sometimes stronger compared to the correlation with the peak and average contact forces. Using higher resolution T1ρ and T2 images, future research can possibly reveal more regional relations between loading and relaxation times and differentiate between the different cartilage layers.

The results of the present study indicate that cartilage is indeed responsive to mechanical stimuli during gait. However, it should be noted that the correlations between cartilage structural or biochemical parameters and loading variables are rather low. This implies that a large portion of the variability in thickness, proteoglycan concentration or collagen concentration and organization is determined by other factors such as genetics, age and geometric characteristics of the joint[6,54]. By considering other frequent activities of daily living (e.g. rising from a chair and stair ascending) of which some may impose higher knee loading, a more exhaustive relation between cartilage thickness distribution and composition and local pressure distribution may be found. As these motions load other regions of the condyles than gait and thus may provide an additional local stimulus for cartilage remodeling.

Nonetheless this study contributes to the current understanding of how cartilage thickness and composition is related to joint loading during walking, the results should be interpreted with respect to its limitations. First, the current analysis used a generic knee model. In future work, the effect of subject-specific detail on cartilage pressure should be explored by including subject-specific cartilage geometries in the knee model. This way the effect of subject-specific detail on cartilage pressure, now lost by using a uniform scaled generic geometry, could be better accounted for. However, currently this methodological adaptation is not yet feasible. Secondly, registration of the subject-specific MRI-based mesh to the generic mesh of the musculoskeletal model will have reduced spatial resolution that might have weakened the calculated correlations between the local thickness and pressure. Third, not all relations between cartilage thickness and biochemical composition and cartilage loading reached significance, but most of their relations support the observed findings. However, due to the large amount of calculated correlations in this exploratory study, one should be careful not over interpreting these results as some may be the results of chance and may not reflect causality.

In conclusion, we found that in a cohort of healthy adults thicker cartilage is associated with higher cartilage loading during walking on a compartmental level. Proteoglycan concentration, estimated using T1ρ mapping, was correlated with loading with increased proteoglycan concentration being related to higher compressive forces but, decreased proteoglycan concentration being related to higher shear forces. Finally, collagen content and organization, estimated using T2 mapping, was correlated with loading, with increased collagen concentration and organization being related to higher pressures and compressive forces.

## Supporting Information

S1 Fig Extended Plug-in-Gait markerset. The full-body extended Plug-in-Gait marker set used during the motion capture. Additional to the original full-body Plug-in-Gait marker set, this marker set is comprised of three-marker clusters on the upper and lower arms and legs and anatomical markers on the sacrum, medial femur epicondyles and the medial malleoli, resulting in a total of 65 markers. Markers in red are the original Plug-in-Gait markers. Green markers are the additionally placed markers.
(EPS)

S2 Fig Correlations between medial thickness and loading. Scatterplots of the significant correlations between mean medial thickness and (A) first peak total knee anterior-posterior contact force, (B) first peak total knee compressional contact force, (C) second peak total knee compressional contact force, (D) second peak total knee resultant contact force, (E) average total pressure during stance, (F) second peak medial compressional contact force, (G) second peak medial resultant contact force and (H) average medial pressure during stance. Between peak medial thickness and (I) second peak total knee compressional contact force, (J) second peak total knee resultant contact force, (K) average total knee pressure during stance, (L) second peak medial compressional contact force, (M) second peak medial resultant contact force and (N) average medial pressure during stance.
(TIF)

S3 Fig Correlations between lateral thickness and loading. Scatterplots of the significant correlations between mean lateral thickness and (A) second peak total knee compressional contact force, (B) second peak total knee resultant contact force, (C) average total knee pressure during stance, (D) Lateral compressional impulse, (E) lateral medial-lateral impulse, (F) lateral resultant impulse and (G) average later pressure during stance. Between peak lateral thickness and (H) first peak total knee anterior-posterior contact force, (I) first peak lateral mean pressure, (J) fist peak lateral maximum pressure, (K) lateral anterior-posterior impulse, (L) lateral compressional impulse, (M) lateral resultant impulse and (N) average lateral pressure during stance.
(TIF)

S4 Fig Correlations between whole joint T1ρ and T2 relaxation time and loading variables. Scatterplots of the significant correlations between the average total T1ρ relaxation time and (A) second peak total knee anterior-posterior contact force, (B) second peak total knee compressional contact force, (C) second peak total knee resultant contact force, (D) total anterior-posterior impulse, (E) total compressional impulse and (F) total resultant impulse. Between the average total T2 relaxation time and (G) second peak total knee compressional contact force, (H) second peak total knee resultant contact force, (I) average total knee pressure during stance.
(TIF)

S5 Fig Correlations between medial and lateral condyle T1ρ relaxation time and loading variables. Scatterplots of the significant correlations between the average medial T1ρ relaxation time and (A) first peak total knee anterior-posterior contact force, (B) second peak medial knee medial-lateral contact force, (C) medial anterior-posterior impulse and (D) medial medial-lateral impulse. Between the average lateral T1ρ relaxation time and (E) second peak total knee anterior-posterior contact force, (F) second peak total knee compressional contact force, (G) second peak total knee resultant contact force, (H) second peak lateral anterior-posterior contact force and (I) second peak lateral medial-lateral contact force.
(TIF)

S1 Table All calculated correlations with total knee loading. (DOCX)

S2 Table All calculated correlations with medial knee loading. (DOCX)

S3 Table All calculated correlations with lateral knee loading. (DOCX)

S4 Table Cartilage parameters for each subject. (DOCX)

S1 Text Registration of subject-specific cartilage mesh on the generic mesh. (DOCX)
