# Supplementary figures and images for: Knee Cartilage Thickness, T1ρ and T2 Relaxation Time Are Related to Articular Cartilage Loading in Healthy Adults
Source: PLoS One. 2017 Jan 11;12(1):e0170002. doi: 10.1371/journal.pone.0170002 (PMC5226797; doi:10.1371/journal.pone.0170002)

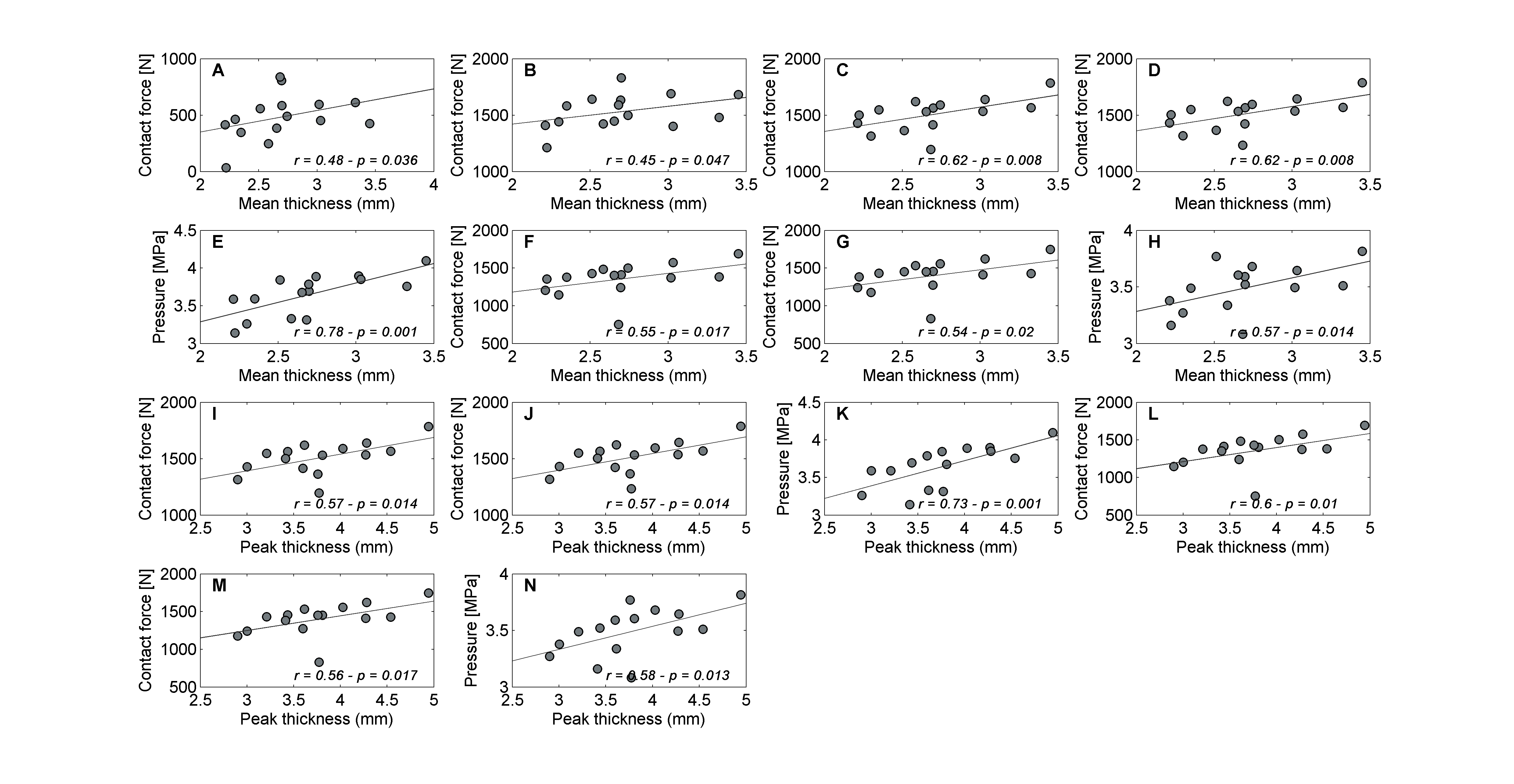

Supplement: S2 Fig — Scatterplots of the significant correlations between mean medial thickness and (A) first peak total knee anterior-posterior contact force, (B) first peak total knee compressional contact force, (C) second peak total knee compressional contact force, (D) second peak total knee resultant contact force, (E) average total pressure during stance, (F) second peak medial compressional contact force, (G) second peak medial resultant contact force and (H) average medial pressure during stance. Between peak medial thickness and (I) second peak total knee compressional contact force, (J) second peak total knee resultant contact force, (K) average total knee pressure during stance, (L) second peak medial compressional contact force, (M) second peak medial resultant contact force and (N) average medial pressure during stance. (TIF) [file pone.0170002.s002.tif]

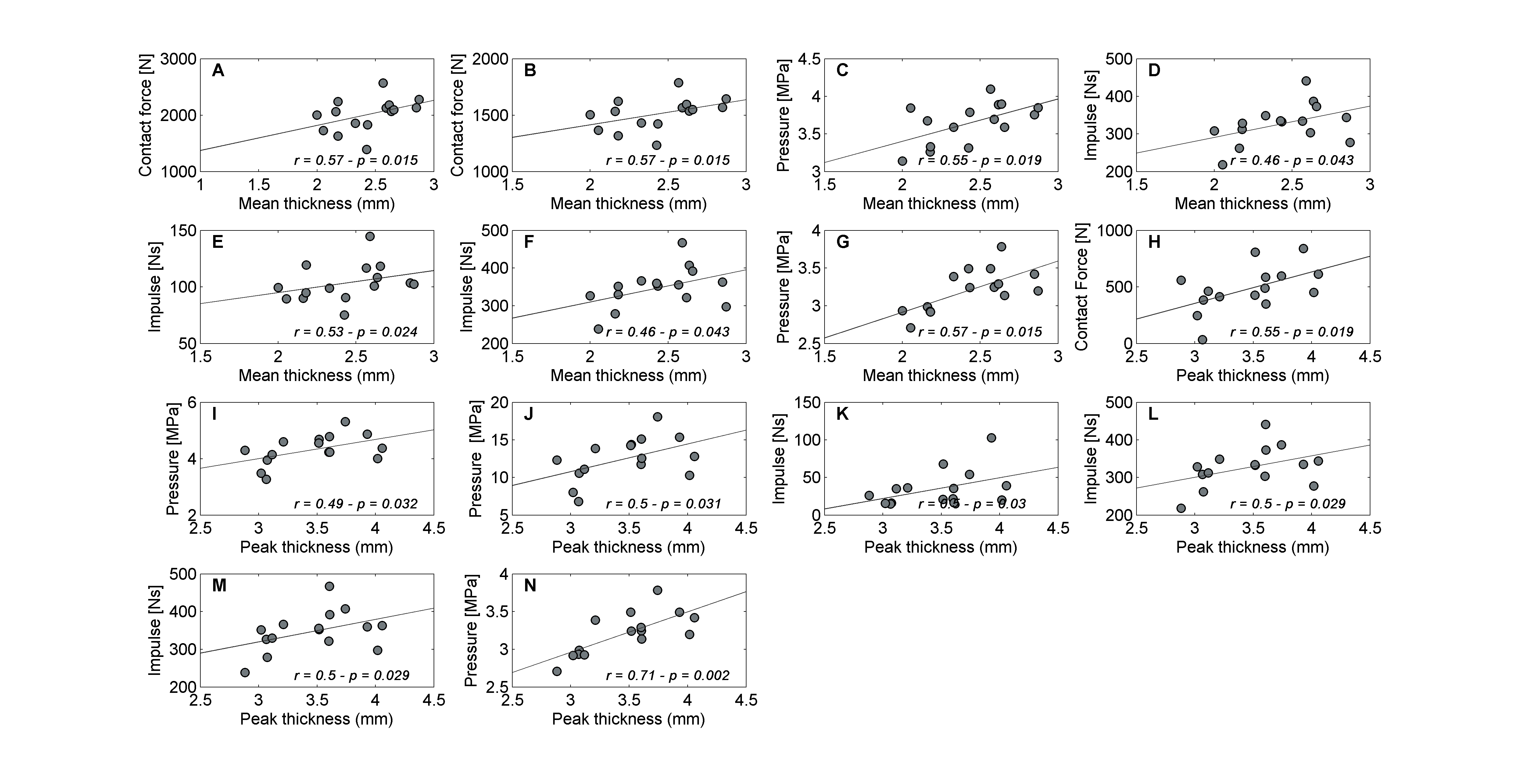

Supplement: S3 Fig — Scatterplots of the significant correlations between mean lateral thickness and (A) second peak total knee compressional contact force, (B) second peak total knee resultant contact force, (C) average total knee pressure during stance, (D) Lateral compressional impulse, (E) lateral medial-lateral impulse, (F) lateral resultant impulse and (G) average later pressure during stance. Between peak lateral thickness and (H) first peak total knee anterior-posterior contact force, (I) first peak lateral mean pressure, (J) fist peak lateral maximum pressure, (K) lateral anterior-posterior impulse, (L) lateral compressional impulse, (M) lateral resultant impulse and (N) average lateral pressure during stance. (TIF) [file pone.0170002.s003.tif]

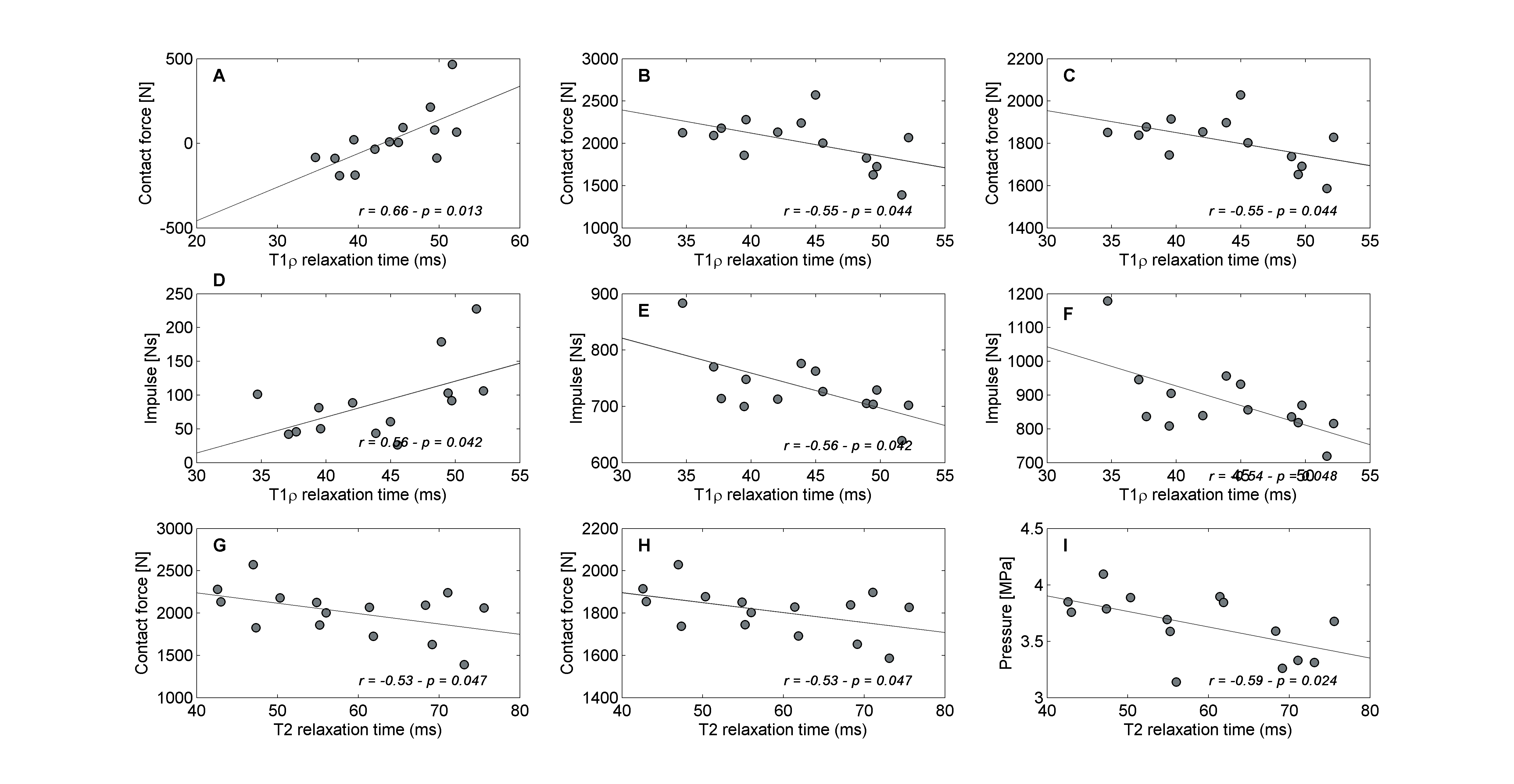

Supplement: S4 Fig — Scatterplots of the significant correlations between the average total T1ρ relaxation time and (A) second peak total knee anterior-posterior contact force, (B) second peak total knee compressional contact force, (C) second peak total knee resultant contact force, (D) total anterior-posterior impulse, (E) total compressional impulse and (F) total resultant impulse. Between the average total T2 relaxation time and (G) second peak total knee compressional contact force, (H) second peak total knee resultant contact force, (I) average total knee pressure during stance. (TIF) [file pone.0170002.s004.tif]

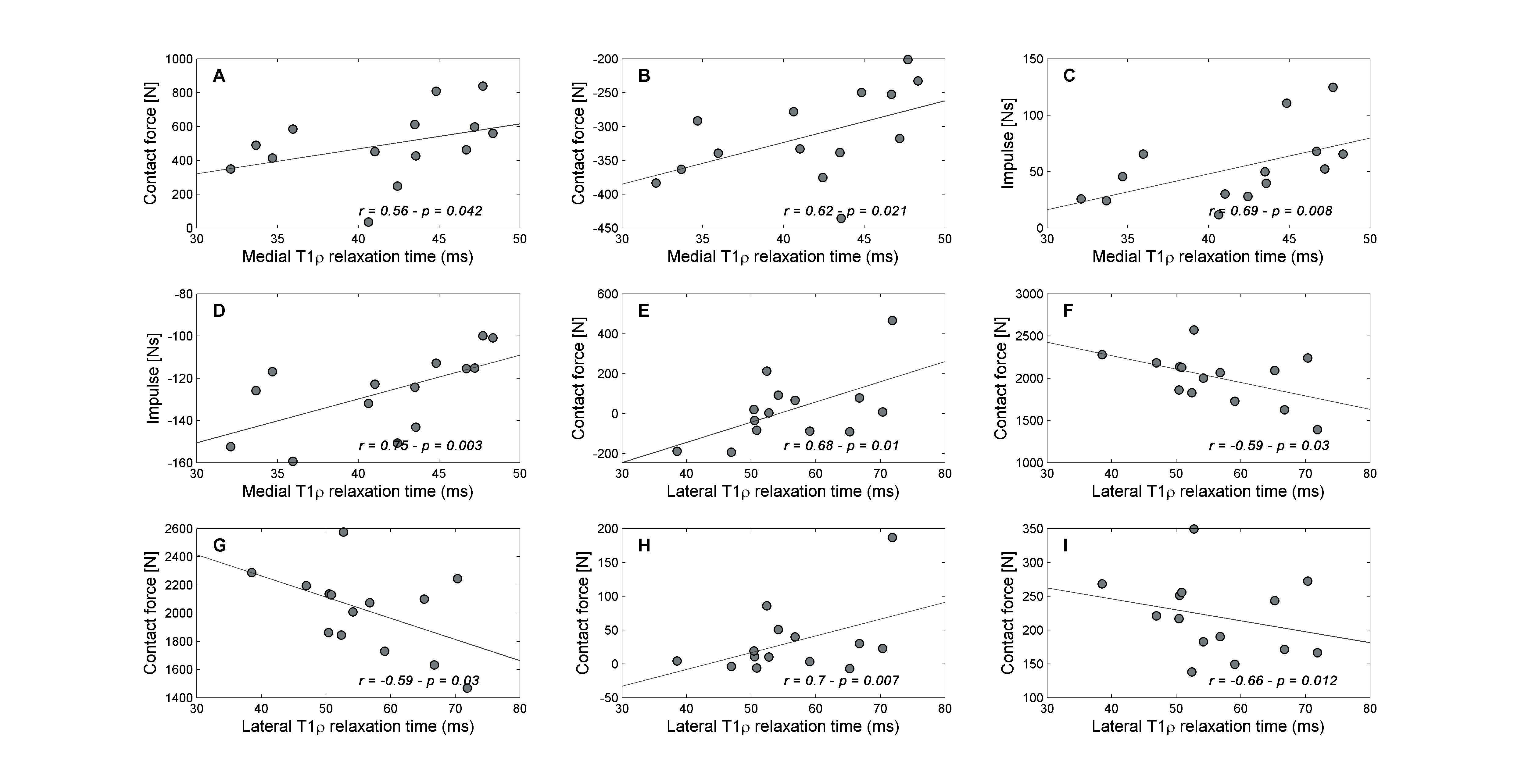

Supplement: S5 Fig — Scatterplots of the significant correlations between the average medial T1ρ relaxation time and (A) first peak total knee anterior-posterior contact force, (B) second peak medial knee medial-lateral contact force, (C) medial anterior-posterior impulse and (D) medial medial-lateral impulse. Between the average lateral T1ρ relaxation time and (E) second peak total knee anterior-posterior contact force, (F) second peak total knee compressional contact force, (G) second peak total knee resultant contact force, (H) second peak lateral anterior-posterior contact force and (I) second peak lateral medial-lateral contact force. (TIF) [file pone.0170002.s005.tif]
