# Supplementary material for: Knee Cartilage Thickness, T1ρ and T2 Relaxation Time Are Related to Articular Cartilage Loading in Healthy Adults
Source: PLoS One. 2017 Jan 11;12(1):e0170002. doi: 10.1371/journal.pone.0170002 (PMC5226797; doi:10.1371/journal.pone.0170002)
Supplement: S1 Table — (DOCX) [file pone.0170002.s006.docx]

**S3. All calculated correlations.**

All calculated correlations between the unnormalized total joint loading variables and the cartilage thickness and T1ρ and T2 relaxation time of the total tibiofemoral and of the medial and lateral condyle.

|  |  |  |  |  |  |  |  |  |  |  |
| --- | --- | --- | --- | --- | --- | --- | --- | --- | --- | --- |
| **Total knee** | Mean medial thickness | Peak medial thickness | Mean lateral thickness | Peak lateral thickness | Mean total T1rho time | Mean total T2 time | Mean medial T1rho time | Mean medial T2 time | Mean lateral T1rho time | Mean lateral T2 time |
| First Peak Contact Force |  |  |  |  |  |  |  |  |  |  |
| *Anterior-Posterior* | 0.48 (0.036)** | 0.3 (0.135) | 0.34 (0.108) | 0.55 (0.019)** | 0.37 (0.192) | -0.24 (0.397) | 0.56 (0.042)** | -0.24 (0.389) | 0.33 (0.253) | -0.16 (0.558) |
| *Compression* | 0.45 (0.047)** | 0.26 (0.177) | 0.24 (0.195) | 0.22 (0.217) | 0.18 (0.542) | -0.05 (0.853) | 0.36 (0.203) | -0.06 (0.822) | 0.28 (0.333) | 0.08 (0.783) |
| *Medial-Lateral* | 0.22 (0.213) | 0.19 (0.249) | 0.14 (0.31) | 0.26 (0.17) | 0.24 (0.417) | -0.02 (0.944) | 0.4 (0.16) | 0.07 (0.802) | 0.11 (0.704) | -0.1 (0.734) |
| *Resultant* | 0.41 (0.065)* | 0.17 (0.275) | 0.26 (0.17) | 0.26 (0.17) | 0.16 (0.573) | -0.1 (0.714) | 0.33 (0.246) | -0.11 (0.686) | 0.29 (0.318) | 0.01 (0.964) |
| Second Peak Contact Force |  |  |  |  |  |  |  |  |  |  |
| *Anterior-Posterior* | -0.28 (0.849) | -0.34 (0.895) | -0.37 (0.913) | -0.09 (0.628) | 0.66 (0.013)** | 0.21 (0.442) | 0.49 (0.075)* | 0.09 (0.753) | 0.68 (0.01)** | 0.3 (0.271) |
| *Compression* | 0.62 (0.008)** | 0.57 (0.014)** | 0.57 (0.015)** | 0.25 (0.187) | -0.55 (0.044)** | -0.53 (0.047)** | -0.47 (0.09)* | -0.43 (0.113) | -0.59 (0.03)** | -0.44 (0.103) |
| *Medial-Lateral* | -0.11 (0.652) | -0.07 (0.604) | -0.18 (0.738) | 0.06 (0.416) | 0.18 (0.532) | -0.03 (0.934) | 0.35 (0.227) | 0.02 (0.954) | -0.11 (0.704) | -0.04 (0.883) |
| *Resultant* | 0.62 (0.008)** | 0.57 (0.014)** | 0.57 (0.015)** | 0.25 (0.187) | -0.55 (0.044)** | -0.53 (0.047)** | -0.47 (0.09)* | -0.43 (0.113) | -0.59 (0.03)** | -0.44 (0.103) |
| Impulse |  |  |  |  |  |  |  |  |  |  |
| *Anterior-Posterior* | 0.22 (0.213) | 0.04 (0.441) | 0.07 (0.406) | 0.31 (0.132) | 0.56 (0.042)** | -0.01 (0.985) | 0.71 (0.006)** | -0.01 (0.964) | 0.49 (0.075)* | 0.06 (0.832) |
| *Compression* | 0.18 (0.262) | 0.06 (0.421) | 0.18 (0.258) | -0.14 (0.69) | -0.56 (0.042)** | -0.25 (0.368) | -0.41 (0.15) | -0.22 (0.434) | -0.51 (0.064)* | -0.15 (0.584) |
| *Medial-Lateral* | 0.15 (0.301) | 0.07 (0.406) | 0.1 (0.357) | 0.15 (0.301) | 0.24 (0.417) | -0.18 (0.524) | 0.41 (0.15) | -0.09 (0.753) | -0.01 (0.988) | -0.27 (0.327) |
| *Resultant* | 0.19 (0.245) | 0.04 (0.441) | 0.23 (0.206) | -0.08 (0.619) | -0.54 (0.048)** | -0.29 (0.289) | -0.38 (0.181) | -0.26 (0.34) | -0.52 (0.062)* | -0.18 (0.524) |
| Mean Pressure |  |  |  |  |  |  |  |  |  |  |
| *First Peak* | 0.43 (0.055)* | 0.26 (0.173) | 0.2 (0.237) | 0.32 (0.121) | 0.3 (0.302) | -0.14 (0.63) | 0.46 (0.101) | -0.14 (0.63) | 0.31 (0.288) | -0.05 (0.863) |
| *Second Peak* | 0.3 (0.141) | 0.44 (0.05)** | -0.02 (0.528) | -0.17 (0.734) | 0.28 (0.333) | -0.4 (0.145) | 0.09 (0.762) | -0.45 (0.097)* | 0.27 (0.349) | -0.28 (0.307) |
| Max Pressure |  |  |  |  |  |  |  |  |  |  |
| *First Peak* | 0.31 (0.132) | 0.24 (0.198) | 0.03 (0.462) | 0.15 (0.301) | 0.34 (0.24) | 0.11 (0.695) | 0.49 (0.081)* | 0.13 (0.658) | 0.31 (0.281) | 0.19 (0.49) |
| *Second Peak* | 0.16 (0.283) | 0.28 (0.157) | -0.02 (0.533) | -0.17 (0.73) | 0.3 (0.295) | -0.14 (0.611) | 0.13 (0.671) | -0.25 (0.361) | 0.42 (0.132) | 0.06 (0.832) |
| Average pressure during stance | 0.78 (0.001)** | 0.73 (0.001)** | 0.55 (0.019)** | 0.31 (0.127) | -0.03 (0.916) | -0.59 (0.024)** | 0.09 (0.75) | -0.51 (0.052)* | -0.07 (0.82) | -0.48 (0.076)* |

nc: not calculated, *p-value <0.10 and ** p-value <0.05
