# Supplementary material for: Knee Cartilage Thickness, T1ρ and T2 Relaxation Time Are Related to Articular Cartilage Loading in Healthy Adults
Source: PLoS One. 2017 Jan 11;12(1):e0170002. doi: 10.1371/journal.pone.0170002 (PMC5226797; doi:10.1371/journal.pone.0170002)
Supplement: S2 Table — (DOCX) [file pone.0170002.s007.docx]

**S3. All calculated correlations.**

All calculated correlations between the unnormalized medial condyle loading variables and the cartilage thickness and T1ρ and T2 relaxation time of the medial condyle.

| **Medial Condyle** | Mean medial thickness | Peak medial thickness | Mean lateral thickness | Peak lateral thickness | Mean total T1rho time | Mean total T2 time | Mean medial T1rho time | Mean medial T2 time | Mean lateral T1rho time | Mean lateral T2 time |
| --- | --- | --- | --- | --- | --- | --- | --- | --- | --- | --- |
| First Peak Contact Force |  |  |  |  |  |  |  |  |  |  |
| *Anterior-Posterior* | 0.42 (0.061)* | 0.24 (0.195) | nc | nc | nc | nc | 0.52 (0.062)* | -0.21 (0.442) | nc | nc |
| *Compression* | 0.16 (0.288) | 0.05 (0.426) | nc | nc | nc | nc | 0.05 (0.856) | 0.06 (0.832) | nc | nc |
| *Medial-Lateral* | -0.35 (0.899) | -0.04 (0.559) | nc | nc | nc | nc | 0.09 (0.773) | 0.26 (0.354) | nc | nc |
| *Resultant* | 0.21 (0.225) | 0.08 (0.391) | nc | nc | nc | nc | 0.14 (0.638) | 0.04 (0.883) | nc | nc |
| Second Peak Contact Force |  |  |  |  |  |  |  |  |  |  |
| *Anterior-Posterior* | -0.26 (0.827) | -0.35 (0.899) | nc | nc | nc | nc | 0.45 (0.104) | 0.08 (0.773) | nc | nc |
| *Compression* | 0.55 (0.017)** | 0.6 (0.01)** | nc | nc | nc | nc | -0.24 (0.409) | -0.34 (0.216) | nc | nc |
| *Medial-Lateral* | -0.34 (0.895) | -0.3 (0.862) | nc | nc | nc | nc | 0.62 (0.021)** | 0.09 (0.763) | nc | nc |
| *Resultant* | 0.54 (0.02)** | 0.56 (0.017)** | nc | nc | nc | nc | -0.31 (0.273) | -0.29 (0.301) | nc | nc |
| Impulse |  |  |  |  |  |  |  |  |  |  |
| *Anterior-Posterior* | 0.09 (0.372) | -0.09 (0.628) | nc | nc | nc | nc | 0.69 (0.008)** | 0.07 (0.812) | nc | nc |
| *Compression* | 0.05 (0.431) | 0.03 (0.467) | nc | nc | nc | nc | -0.24 (0.409) | -0.04 (0.893) | nc | nc |
| *Medial-Lateral* | -0.09 (0.623) | 0.09 (0.377) | nc | nc | nc | nc | 0.75 (0.003)** | 0.23 (0.411) | nc | nc |
| *Resultant* | 0.03 (0.457) | 0 (0.508) | nc | nc | nc | nc | -0.23 (0.426) | -0.03 (0.913) | nc | nc |
| Mean Pressure |  |  |  |  |  |  |  |  |  |  |
| *First Peak* | 0.39 (0.076)* | 0.26 (0.177) | nc | nc | nc | nc | 0.43 (0.128) | -0.18 (0.532) | nc | nc |
| *Second Peak* | 0.11 (0.343) | 0.23 (0.202) | nc | nc | nc | nc | 0 (1) | -0.31 (0.259) | nc | nc |
| Max Pressure |  |  |  |  |  |  |  |  |  |  |
| *First Peak* | 0.24 (0.191) | 0.15 (0.301) | nc | nc | nc | nc | 0.35 (0.221) | 0.05 (0.853) | nc | nc |
| *Second Peak* | 0.17 (0.275) | 0.25 (0.184) | nc | nc | nc | nc | 0.05 (0.88) | -0.32 (0.237) | nc | nc |
| Average pressure during stance | 0.57 (0.014)** | 0.58 (0.013)** | nc | nc | nc | nc | 0 (1) | -0.47 (0.078)* | nc | nc |

nc: not calculated, *p-value <0.10 and ** p-value <0.05
