# Supplementary material for: Knee Cartilage Thickness, T1ρ and T2 Relaxation Time Are Related to Articular Cartilage Loading in Healthy Adults
Source: PLoS One. 2017 Jan 11;12(1):e0170002. doi: 10.1371/journal.pone.0170002 (PMC5226797; doi:10.1371/journal.pone.0170002)
Supplement: S3 Table — (DOCX) [file pone.0170002.s008.docx]

**S3. All calculated correlations.**

All calculated correlations between the unnormalized lateral condyle loading variables and the cartilage thickness and T1ρ and T2 relaxation time of the lateral condyle.

| **Lateral Condyle** | Mean medial thickness | Peak medial thickness | Mean lateral thickness | Peak lateral thickness | Mean total T1rho time | Mean total T2 time | Mean medial T1rho time | Mean medial T2 time | Mean lateral T1rho time | Mean lateral T2 time |
| --- | --- | --- | --- | --- | --- | --- | --- | --- | --- | --- |
| First Peak Contact Force |  |  |  |  |  |  |  |  |  |  |
| *Anterior-Posterior* | nc | nc | 0.23 (0.209) | 0.38 (0.08)* | nc | nc | nc | nc | 0.25 (0.382) | -0.29 (0.289) |
| *Compression* | nc | nc | -0.16 (0.721) | -0.37 (0.913) | nc | nc | nc | nc | 0.24 (0.417) | -0.14 (0.621) |
| *Medial-Lateral* | nc | nc | -0.42 (0.942) | -0.39 (0.928) | nc | nc | nc | nc | 0.21 (0.464) | -0.16 (0.576) |
| *Resultant* | nc | nc | -0.11 (0.657) | -0.27 (0.843) | nc | nc | nc | nc | 0.25 (0.382) | -0.13 (0.639) |
| Second Peak Contact Force |  |  |  |  |  |  |  |  |  |  |
| *Anterior-Posterior* | nc | nc | -0.32 (0.879) | -0.05 (0.569) | nc | nc | nc | nc | 0.7 (0.007)** | 0.21 (0.45) |
| *Compression* | nc | nc | 0.29 (0.144) | -0.05 (0.574) | nc | nc | nc | nc | -0.49 (0.075)* | -0.2 (0.474) |
| *Medial-Lateral* | nc | nc | -0.39 (0.926) | -0.06 (0.594) | nc | nc | nc | nc | -0.66 (0.012)** | -0.21 (0.442) |
| *Resultant* | nc | nc | 0.32 (0.121) | -0.02 (0.533) | nc | nc | nc | nc | -0.52 (0.062)* | -0.24 (0.397) |
| Impulse |  |  |  |  |  |  |  |  |  |  |
| *Anterior-Posterior* | nc | nc | 0.27 (0.16) | 0.5 (0.03)** | nc | nc | nc | nc | 0.35 (0.227) | -0.16 (0.558) |
| *Compression* | nc | nc | 0.46 (0.043)** | 0.5 (0.029)** | nc | nc | nc | nc | -0.09 (0.773) | -0.11 (0.695) |
| *Medial-Lateral* | nc | nc | 0.53 (0.024)** | 0.24 (0.198) | nc | nc | nc | nc | -0.5 (0.072)* | -0.27 (0.333) |
| *Resultant* | nc | nc | 0.46 (0.043)** | 0.5 (0.029)** | nc | nc | nc | nc | -0.09 (0.773) | -0.11 (0.695) |
| Mean Pressure |  |  |  |  |  |  |  |  |  |  |
| *First Peak* | nc | nc | 0.35 (0.098)* | 0.49 (0.032)** | nc | nc | nc | nc | 0.26 (0.366) | -0.15 (0.602) |
| *Second Peak* | nc | nc | 0.19 (0.249) | 0.36 (0.096)* | nc | nc | nc | nc | 0.28 (0.333) | -0.1 (0.724) |
| Max Pressure |  |  |  |  |  |  |  |  |  |  |
| *First Peak* | nc | nc | 0.36 (0.093)* | 0.5 (0.031)** | nc | nc | nc | nc | 0.27 (0.357) | -0.08 (0.783) |
| *Second Peak* | nc | nc | 0.06 (0.416) | 0.2 (0.233) | nc | nc | nc | nc | 0.43 (0.124) | -0.05 (0.853) |
| Average pressure during stance | nc | nc | 0.57 (0.015)** | 0.71 (0.002)** | nc | nc | nc | nc | 0.09 (0.773) | -0.33 (0.232) |

nc: not calculated, *p-value <0.10 and ** p-value <0.05
