# Supplementary material for: Knee Cartilage Thickness, T1ρ and T2 Relaxation Time Are Related to Articular Cartilage Loading in Healthy Adults
Source: PLoS One. 2017 Jan 11;12(1):e0170002. doi: 10.1371/journal.pone.0170002 (PMC5226797; doi:10.1371/journal.pone.0170002)
Supplement: S4 Table — (DOCX) [file pone.0170002.s009.docx]

**S5. Cartilage parameters for each subject**

Supplementary table 4:

Table presenting the individual datapoints used for the correlation analysis.

|  | **T1ρ relaxation time** | | | **T2 relaxation time** | | | **Mean thickness** | | **Peak thickness** | |
| --- | --- | --- | --- | --- | --- | --- | --- | --- | --- | --- |
|  | Total | Medial | Lateral | Total | Medial | Lateral | Medial | Lateral | Medial | Lateral |
| Subject 1 | 42.05 | 43.49 | 40.72 | 43.01 | 35.51 | 50.50 | 3.33 | 2.85 | 4.54 | 4.06 |
| Subject 2 | 34.70 | 35.96 | 33.43 | 54.86 | 58.89 | 50.84 | 2.70 | 2.59 | 3.44 | 3.61 |
| Subject 3 | 37.69 | 33.67 | 41.71 | 50.31 | 53.68 | 46.95 | 2.74 | 2.62 | 4.03 | 3.60 |
| Subject 4 | 48.92 | 44.83 | 53.02 | 47.34 | 42.28 | 52.40 | 2.70 | 2.43 | 3.60 | 3.52 |
| Subject 5 | 52.18 | 47.19 | 57.17 | 61.39 | 66.67 | 56.79 | 3.02 | 2.64 | 4.27 | 3.74 |
| Subject 6 | 39.45 | 34.68 | 39.81 | 55.24 | 60.07 | 50.41 | 2.21 | 2.33 | 3.00 | 3.21 |
| Subject 7 | 78.03 | 78.08 | 77.99 | 75.57 | 76.59 | 74.55 | 2.65 | 2.16 | 3.81 | 3.07 |
| Subject 8 | 45.54 | 40.63 | 50.44 | 56.01 | 57.84 | 54.18 | 2.22 | 2.00 | 3.41 | 3.07 |
| Subject 9 | 49.44 | 46.69 | 52.20 | 69.15 | 68.21 | 66.76 | 2.30 | 2.18 | 2.90 | 3.12 |
| Subject 10 | 39.59 | 41.03 | 38.16 | 42.60 | 46.68 | 38.52 | 3.03 | 2.87 | 4.28 | 4.02 |
| Subject 11 | 44.98 | 43.56 | 46.40 | 46.99 | 41.26 | 52.71 | 3.45 | 2.57 | 4.94 | 3.51 |
| Subject 12 | 43.86 | 42.43 | 45.29 | 71.07 | 71.80 | 70.33 | 2.58 | 2.18 | 3.61 | 3.02 |
| Subject 13 | 51.65 | 47.71 | 55.59 | 73.12 | 74.39 | 71.85 | 2.68 | 2.43 | 3.77 | 3.93 |
| Subject 14 | 49.73 | 48.34 | 51.13 | 61.85 | 66.04 | 59.06 | 2.51 | 2.05 | 3.76 | 2.88 |
| Subject 15 | 37.10 | 32.11 | 42.09 | 68.32 | 71.41 | 65.23 | 2.35 | 2.66 | 3.21 | 3.61 |
| Subject 7 was excluded from the T1ρ analysis due to image artefacts. | | | | | |  |  |  |  |  |
|  | | | | | |  |  |  |  |  |
