# Supplementary material for: Knee Cartilage Thickness, T1ρ and T2 Relaxation Time Are Related to Articular Cartilage Loading in Healthy Adults
Source: PLoS One. 2017 Jan 11;12(1):e0170002. doi: 10.1371/journal.pone.0170002 (PMC5226797; doi:10.1371/journal.pone.0170002)
Supplement: S1 Text — (DOCX) [file pone.0170002.s010.docx]

**S2. Registration of subject-specific cartilage mesh on the generic mesh.**

The subject-specific thickness maps were anisotropically registered to the generic cartilage mesh used in the musculoskeletal model. Therefore, the generic mesh was scaled non-uniformly to better match the size of the subject-specific mesh. Next, both meshes were positioned onto each other using an iterative closest point algorithm. Subsequently, for all faces of the subject-specific mesh the intersection point of a line through the face center in face normal direction and the generic mesh was determined. Last, the subject-specific thickness of the face was assigned to the intersected face of the generic mesh.
